# Identification of key genes for cuproptosis in carotid atherosclerosis

**DOI:** 10.3389/fcvm.2024.1471153

**Published:** 2024-11-01

**Authors:** Xize Wu, Jian Kang, Xue Pan, Chentian Xue, Jiaxiang Pan, Chao Quan, Lihong Ren, Lihong Gong, Yue Li

**Affiliations:** ^1^ Department of Critical Care Medicine, Nantong Hospital of Traditional Chinese Medicine, Nantong Hospital Affiliated to Nanjing University of Chinese Medicine, Nantong, Jiangsu, China; ^2^ Graduate School, Liaoning University of Traditional Chinese Medicine, Shenyang, Liaoning, China; ^3^ College of Traditional Chinese Medicine, Dazhou Vocational College of Chinese Medicine, Dazhou, Sichuan, China; ^4^ Graduate School, Nanjing University of Traditional Chinese Medicine, Nanjing, China; ^5^ Department of Cardiology, The Affiliated Hospital of Liaoning University of Traditional Chinese Medicine, Shenyang, Liaoning, China; ^6^ Liaoning Provincial Key Laboratory of TCM Geriatric Cardio-Cerebrovascular Diseases, Shenyang, Liaoning, China

**Keywords:** atherosclerosis, cuproptosis, unsupervised clustering analysis, machine learning model, nomogram

## Abstract

**Background:**

Atherosclerosis is a leading cause of cardiovascular disease worldwide, while carotid atherosclerosis (CAS) is more likely to cause ischemic cerebrovascular events. Emerging evidence suggests that cuproptosis may be associated with an increased risk of atherosclerotic cardiovascular disease. This study aims to explore the potential mechanisms linking cuproptosis and CAS.

**Methods:**

The GSE100927 and GSE43292 datasets were merged to screen for CAS differentially expressed genes (DEGs) and intersected with cuproptosis-related genes to obtain CAS cuproptosis-related genes (CASCRGs). Unsupervised cluster analysis was performed on CAS samples to identify cuproptosis molecular clusters. Weighted gene co-expression network analysis was performed on all samples and cuproptosis molecule clusters to identify common module genes. CAS-specific DEGs were identified in the GSE100927 dataset and intersected with common module genes to obtain candidate hub genes. Finally, 83 machine learning models were constructed to screen hub genes and construct a nomogram to predict the incidence of CAS.

**Results:**

Four ASCRGs (NLRP3, SLC31A2, CDKN2A, and GLS) were identified as regulators of the immune infiltration microenvironment in CAS. CAS samples were identified with two cuproptosis-related molecular clusters with significant biological function differences based on ASCRGs. 220 common module hub genes and 1,518 CAS-specific DEGs were intersected to obtain 58 candidate hub genes, and the machine learning model showed that the Lasso + XGBoost model exhibited the best discriminative performance. Further external validation of single gene differential analysis and nomogram identified SGCE, PCDH7, RAB23, and RIMKLB as hub genes; SGCE and PCDH7 were also used as biomarkers to characterize CAS plaque stability. Finally, a nomogram was developed to assess the incidence of CAS and exhibited satisfactory predictive performance.

**Conclusions:**

Cuproptosis alters the CAS immune infiltration microenvironment and may regulate actin cytoskeleton formation.

## 1 Introduction

Atherosclerosis (AS) is a chronic inflammatory pathological change that occurs in the vascular wall and is characterized by lipid deposition and immune cell infiltration. It serves as the pathological basis for various cardiovascular diseases (CVDs), including ischemic heart disease and stroke (1). AS commonly manifests in the coronary, brain, and carotid arteries. Areas of relatively slow blood flow, rapid dilation of vessel diameters, and vertexing of blood flow at the carotid bifurcation are favored for carotid atherosclerosis (CAS) (2). The global prevalence of CAS in 2020 was as high as 27.6% in people aged 30–90 years, CAS is more likely to cause ischemic cerebrovascular events, which significantly increased the incidence of CVDs (3).

Copper is an essential trace element in the human body and functions as a cofactor for numerous enzymes involved in critical cellular processes, such as transcriptional regulation, oxidoreductase reactions, inflammation, immune function, mitochondrial electron transport, and free radical scavenging (4). Intracellular copper ion content is tightly regulated, and any imbalance can lead to oxidative stress and abnormal cellular autophagy (5, 6). The excessive accumulation of copper ions results in abnormal aggregation of lipoylated proteins, interference with iron-sulfur cluster proteins in the respiratory chain complex, and ultimately induces a protein-toxic stress response leading to cell death. This type of cell death, triggered by copper ion accumulation, is termed “cuproptosis” and can contribute to the onset of various diseases (7). Previous studies have shown that serum copper deficiency promotes the development of AS through increased cholesterol levels, elevated blood pressure, and impaired glucose tolerance (8, 9). However, subsequent research has revealed that elevated serum copper levels are associated with an increased risk of atherosclerotic CVDs, which contradicts the previously reported association between serum copper and CVDs outcomes (10, 11). High serum copper levels accelerate atherosclerotic plaque formation by affecting lipid metabolism, low density lipoprotein oxidation, and inflammation, thereby increasing the risk of atherosclerotic heart disease (12, 13).

Therefore, this study was based on bioinformatics to uncover the underlying mechanisms and pivotal genes associated with cuproptosis in CAS. Employing differential expression analysis to identify CAS cuproptosis-related genes (CASCRGs) and immune profiles in CAS by comparing control and CAS samples. Subsequently, unsupervised cluster analysis on CAS samples, utilizing CASCRGs, aimed to delineate cuproptosis-associated clusters and evaluate the differences in gene expression, immunity, and biological processes among these clusters. Further, candidate hub genes were discerned through the application of weighted gene co-expression network analysis (WGCNA). Multiple prediction models were then developed based on machine learning algorithms. The efficacy of these models was rigorously tested using a nomogram, calibration curves, and decision curve analysis. Additionally, the study incorporated a gender-stratified approach and examined the stability of CAS plaques, thereby enriching the understanding of the disease’s complexity and heterogeneity.

## 2 Materials & methods

### 2.1 Subjects and dataset acquisition

**Figure 1:**
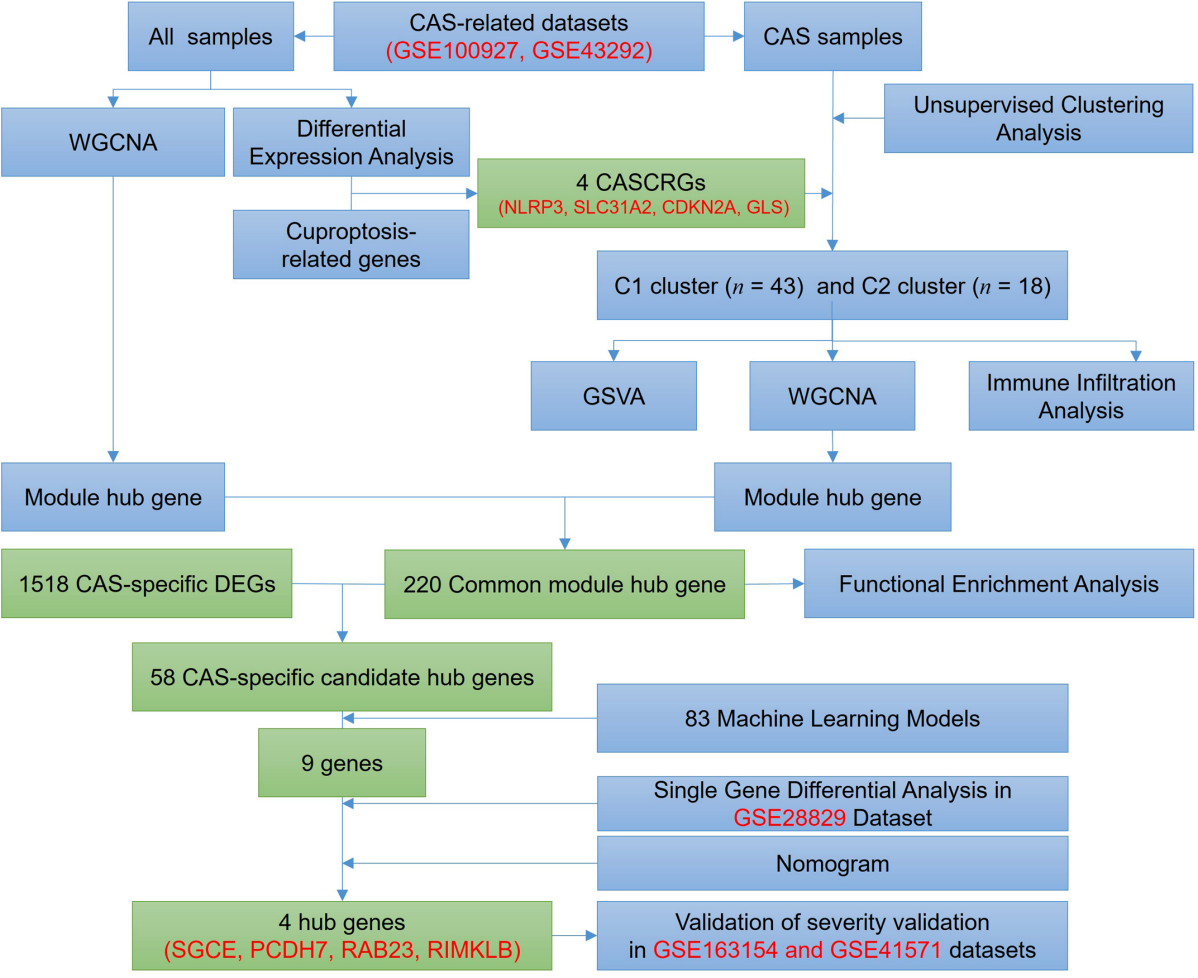
Flow chart of this study.

The entire study process is depicted in Figure 1. Five gene expression profiles [GSE100927 (14), GSE43292 (15), GSE28829 (16), GSE163154 (17), and GSE41571 (18)] related to CAS were retrieved from the Gene Expression Omnibus database (19) (GEO, https://www.ncbi.nlm.nih.gov/geo/) under the keywords “carotid atherosclerosis”. Among them, the GSE28829 dataset was used for external validation, and the GSE163154 and GSE41571 datasets were used to identify CAS stabilized and unstabilized plaques.

### 2.2 Identification of differentially expressed genes (DEGs)

The GSE100927 and GSE43292 datasets were merged and standardized using the “Affy” R package, while batch effects were removed with the “SVA” R package (20, 21). DEGs associated with CAS were identified by comparing disease and control groups using the “limma” R package, with the criteria set at *P* < 0.05 and |logFC|>0.5 (1.4-fold differential expression) for DEG selection (22). From the literature, 50 cuproptosis-related genes (CRGs) were obtained and intersected them with the DEGs of CAS to obtain CASCRGs (7, 23–25).

### 2.3 Unsupervised clustering analysis of CAS samples

Unsupervised clustering analysis of CAS samples based on CASCRGs expression profiles was performed using the “ConsensusClusterPlus” R package (26). The CAS samples were grouped by applying the k-means algorithm with 1,000 iterations, k = 9, seed = 123,456, reps = 50, pItem = 0.8, pFeature = 1, clusterAlg = km, distance = euclidean. The appropriate number of clusters was determined based on the matrix heat map, consistent cumulative distribution function curve, delta area plot, cluster-consensus plot, and item-consensus plot.

### 2.4 Immune infiltration analysis and correlation analysis

The degree of infiltration of 22 immune cells was quantified using the CIBERSORT deconvolution algorithm based on gene microarray data (27). Differences between the two groups (the C1 cluster compared to the C2 cluster; the control sample compared to the CAS sample) were compared using the Wilcox test, and the results were visualized using the “vioplot” package (28). Subsequently, Spearman correlation analysis was employed to reveal the relationship between ASCRGs and immune cells.

### 2.5 Gene set variation analysis (GSVA)

The “GSVA” package was used to conduct a GSVA enrichment analysis for different CRGs clusters, considering a significant change if the |t value of the GSVA score| was greater than 2 (29).

### 2.6 WGCNA

WGCNA was employed to identify co-expression modules by clustering the samples using the “WGCNA” R package. The value of “CutHeight” was set to 60 to remove the outlier samples, and a co-expression network for the gene expression matrices of the remaining samples was constructed. The soft threshold corresponding to fit R^2^ = 0.8 was chosen for the construction of gene modules, while the minimum number of module genes (minSize) was specified to be 10, and the most relevant module for the trait was selected (30).

### 2.7 Functional enrichment analysis

Imported the genes into the David database (https://david.abcc.ncifcrf.gov/) (31) for functional enrichment analysis (32), set *P* < 0.05 as the screening condition.

### 2.8 Gene set enrichment analysis (GSEA)

The “GSEA” R package was used to explore the related pathways of candidate hub genes and to calculate the correlation between candidate hub genes and other genes (33). All genes were then sorted from highest to lowest according to their correlation, and these sorted genes were the set of genes to be tested. The signaling pathway set was called a “predefined set” to detect its enrichment in the gene set.

### 2.9 Construction of predictive model based on multiple machine learning methods

The “randomForestSRC” “glmnet” “plsRglm” gbm “caret” “mboost” “e1071” “BART “MASS” “snowfall” “xgboost” R packages were used to establish 113 machine learning models screening for the hub genes, including the least absolute shrinkage and selection operator (LASSO) regression, random forest model, support vector machine model, generalized linear model and extreme gradient boosting (XGBoost), gradient boosting machine, and so on (34). The merged dataset of GSE100927 and GSE43292 was used as a training set, and the GSE28829 dataset was used as a validation set. The area under the receiver operating characteristic (ROC) curve was visualized using the “pROC” R package (35). F1 scores were calculated based on precision and recall, and then the best models were screened based on AUC values, F1 scores, and gene counts. The optimal machine learning model was identified and externally validated using the Wilcoxon rank-sum test for single gene difference analysis on the GSE28829 dataset.

### 2.10 Construction and validation of a nomogram model

A nomogram was established using the “rms” R package to predict the probability of occurrence of CAS, and its predictive power was estimated by using calibration curves and decision curve analysis.

### 2.11 Statistical analysis

All statistical analyses were performed using R software, and *P* < 0.05 was considered significant.

## 3 Results

### 3.1 Cuproptosis regulator modulates the immune infiltration microenvironment in CAS

**Figure 2:**
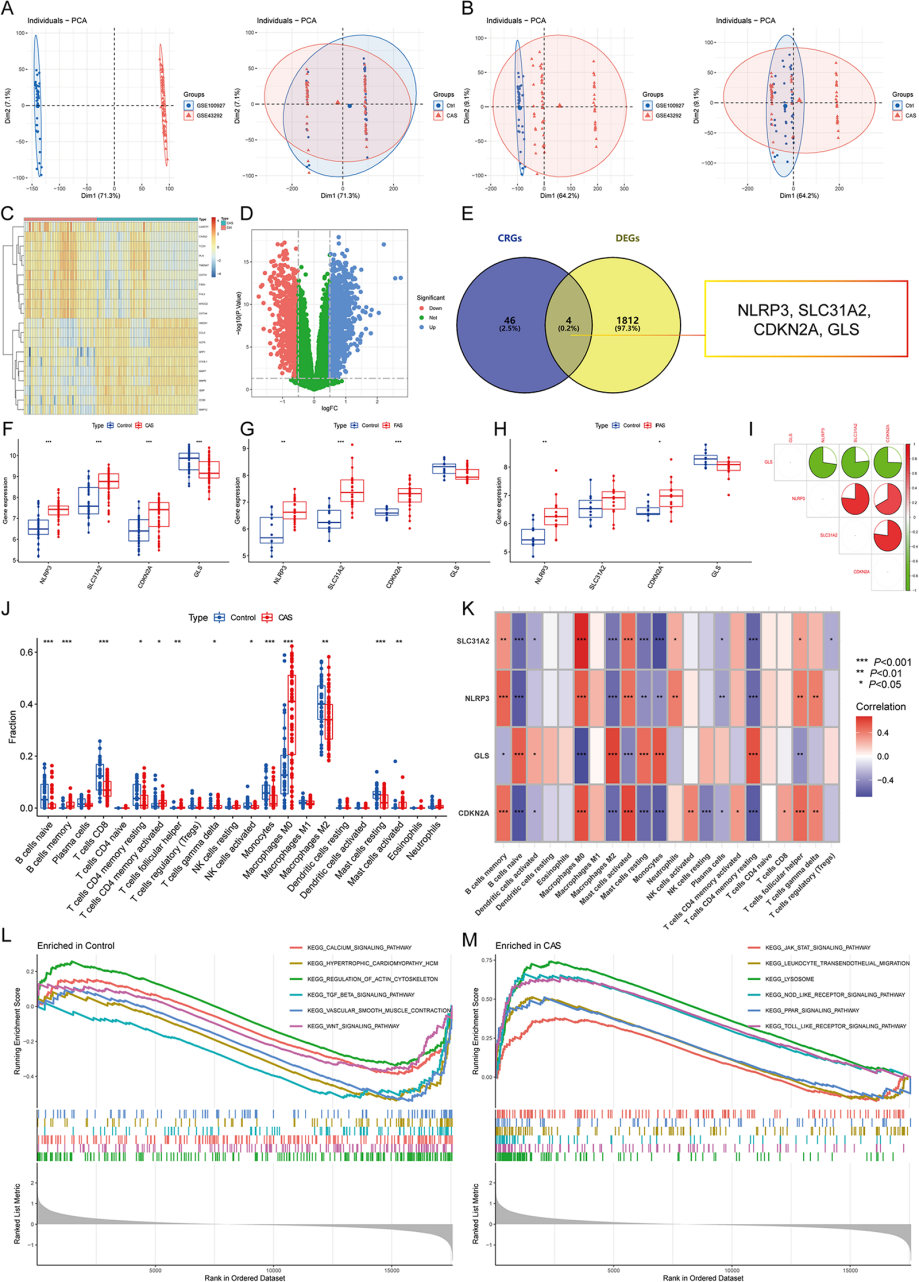
Identification of CAS cuproptosis-related genes (CASCRGs). **(A)** The principal component analysis (PCA) of the two datasets and clinical characteristics; **(B)** The PCA of the combined dataset and clinical characteristics. The horizontal axis represents the first principal component and the vertical axis represents the second principal component. It is seen that the dataset before merging has some differences, and the normal and CAS groups have some similarities. After merging and removing the batch effect, it is seen that the merged dataset has some similarity and the normal and CAS groups have some differences. **(C,D)** The **(C)** heatmap and **(D)** volcano plot for CAS differentially expressed genes (DEG)s. The heatmap demonstrates significantly differentially expressed DEGs in the normal and CAS groups, with red representing high expression and blue representing low expression. The volcano plot demonstrates DEGs with *P* < 0.05 and |logFC|>0.5, where red represents DEGs down-regulated in CAS, while blue represents DEGs up-regulated in CAS, and green represents genes not differentially expressed. **(E)** Venn diagram showing 4 CASCRGs; **(F–H)** The differential expression of CASCRGs in different arterial beds of **(F)** CAS, **(G)** femoral AS (FAS), and **(H)** inflapulite AS (IPAS); **(I)** Correlation analysis between CASCRGs. There was a strong synergy between SLC31A2, NLRP3 and CDKN2A, whereas GLS showed antagonistic effects. **(J)** Boxplot showing differences in immune infiltration between CAS and control groups; **(K)** Correlation analysis of the CORGs with infiltrating immune cells; (L-M) The GSEA for control **(L)** and CAS **(M)** samples. *P < 0.05, **P < 0.01, ***P < 0.001.

The differential expression gene analysis of CAS identified 1,816 DEGs (Figures 2A–D), and 4 CASCRGs (NLRP3, SLC31A2, CDKN2A, and GLS) were obtained by taking the intersection with cuproptosis-related genes, of which NLRP3 (logFC = 0.81), SLC31A2 (logFC = 0.92), and CDKN2A (logFC = 0.71) were highly expressed in CAS samples, while GLS (logFC = −0.51) was lowly expressed (*P* < 0.05 and |logFC|>0.5) (Figures 2E,F). Further investigating whether CASCRGs are specific in CAS, GSE100927 was stratified into CAS, femoral AS (FAS), and infrapopliteal AS (IPAS). The results showed that SLC31A2, NLRP3, and CDKN2A had differential expression in FAS (*P* < 0.05 and |logFC|>0.5); and NLRP3 and CDKN2A in IPAS (*P* < 0.05 and |logFC|>0.5), which suggests that NLRP3 and CDKN2A were differentially expressed in CAS, FAS and IPAS, whereas GLS has reduced specific expression in CAS (Figures 2G,H). Further correlation analyses demonstrated a strong synergistic effect between SLC31A2, NLRP3, and CDKN2A, whereas GLS exhibited antagonistic effects (Figure 2I).

The results of immune infiltration analysis revealed significantly higher levels of memory B cells, activated memory CD4T cells, follicular helper T cells, γ-δ T cells, M0 macrophages, and activated mast cells in CAS (*P* < 0.05). Conversely, significantly lower levels of naive B cells, CD8T cells, resting memory CD4T cells, activated NK cells, monocytes, M2 macrophages, and resting mast cells were observed in CAS (*P* < 0.05) (Figure 2J). Furthermore, correlation analysis demonstrated that CASCRGs were strongly associated with memory B cells, naive B cells, activated dendritic cells, M0 macrophages, M2 macrophages, activated mast cells, resting mast cells, monocytes, plasma cells, resting memory CD4T cells, follicular helper T cells, and γ-δ T cells (*P* < 0.05), suggesting that CASCRGs is expressed in various immune cells of CAS and play a role in regulating the immune infiltration environment in CAS (Figure 2K).

Comprehensive functional enrichment analysis revealed that the common pathogenesis of CAS mainly involves various cardiomyopathies, immune responses, cell migration, and cytokine-mediated signaling pathways, including the JAK-STAT signaling pathway (Figures 2L,M).

### 3.2 Identification of cuproptosis clusters in CAS

**Figure 3:**
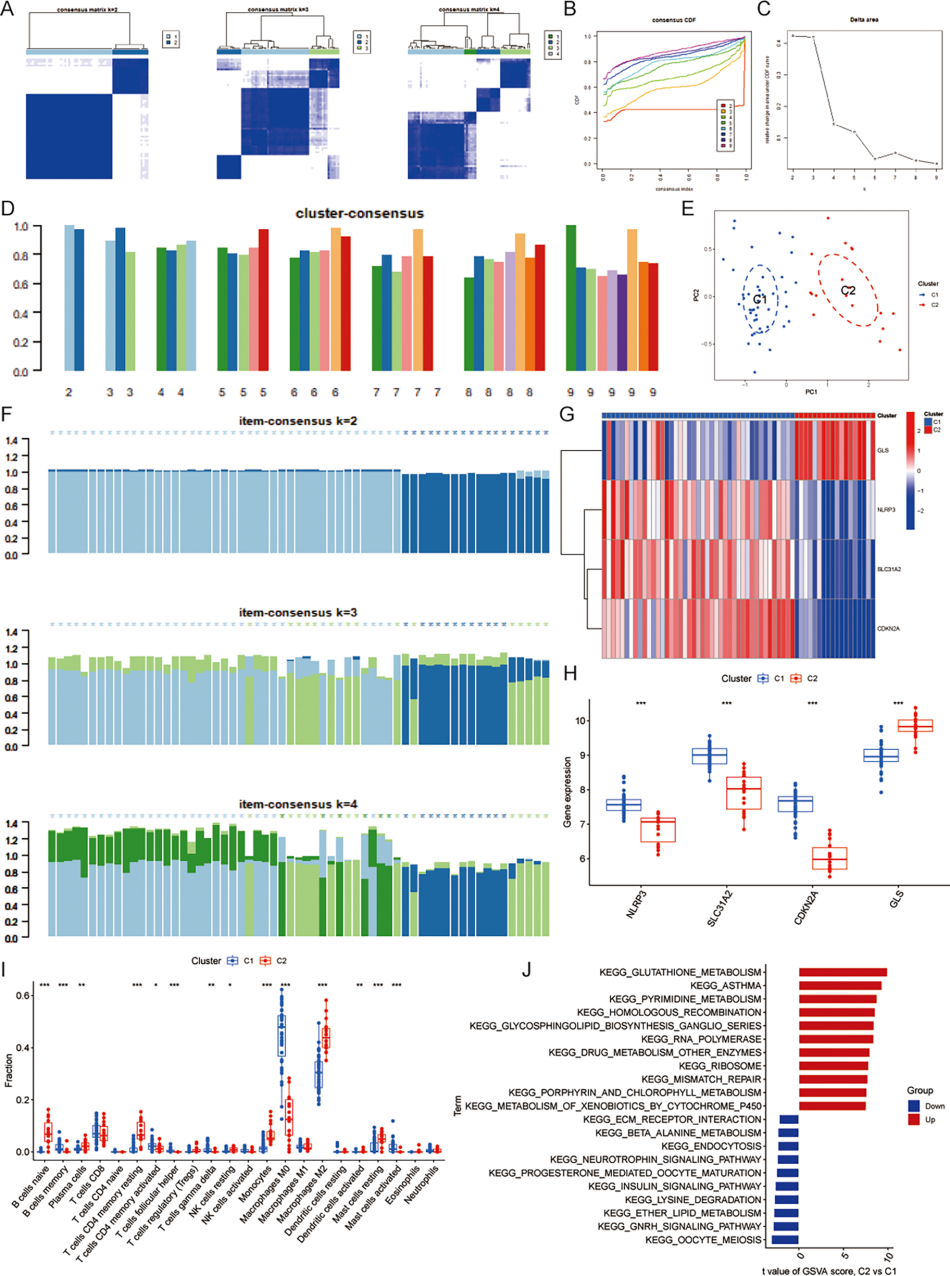
Identification of cuproptosis-related molecular clusters in CAS. **(A)** Consensus clustering matrix; **(B–D)** Representative **(B)** cumulative distribution function (CDF) curves and **(C)** delta area curves, and the **(D)** score of consensus clustering; **(E)** The PCA showing subtype distribution. The horizontal axis represents the first principal component and the vertical axis represents the second principal component. It can be seen that there is some variability between the two clusters. **(F)** The item-consensus plot showing the total item-consensus values and sample purity for each sample. **(G–H)** The **(G)** heatmap and **(H)** boxplot of expression levels of the 4 CASCRGs between the two cuproptosis clusters. The heatmap demonstrates that SLC31A2, NLRP3 and CDKN2A are highly expressed in the C1cluster, while GLS is highly expressed in the C2 cluster. **(I)** Comparison of immune cell infiltration between two clusters; **(J)** The GSVA analysis of the C1 and C2 clusters. *P < 0.05, **P < 0.01.

Based on the matrix heatmap, it can be seen that the CAS samples are clearly divided into 2 clusters with less clutter around them (Figure 3A); the consensus cumulative distribution function and the inflection point method of delta area seem to suggest that k = 4 is better (Figures 3B,C). However, the cluster-consensus plot shows the mean of the pairwise consensus values of the members in that cluster, with a higher mean value representing higher stability, and the results show that k = 2 has the highest mean value, and there is not much difference between the two clusters, which is stable (Figure 3D). In addition, the vertical bar of the item-consensus plot represents each sample, and the height of the bar represents the total item-consensus values of the sample, and the purity of the sample can also be seen, and the results show that, when k = 2, the total item-consensus values of the sample are higher, and the purity is good, while k = 3 and 4, the performance is not as satisfactory as at k = 2. Therefore, k = 2 was used for the subsequent study (Figure 3F). A consensus clustering algorithm was applied to classify the 61 CAS samples based on the expression profiles of the 4 CASCRGs, resulting in two distinct and stable groups: the C1 cluster (*n* = 43) and the C2 cluster (*n* = 18) (Figures 3A–F). Among them, NLRP3, SLC31A2, and CDKN2A were highly expressed in the C1 cluster, while GLS was highly expressed in the C2 cluster (Figures 3G,H). Furthermore, immune infiltration analyses of the two clusters revealed that the C1 cluster had a significantly higher abundance of memory B cells, activated memory CD4T cells, follicular helper T cells, γ-δ T cells, M0 macrophages, and activated mast cells (*P* < 0.05), while the C2 cluster had significantly higher levels of naive B cells, plasma cells, resting memory CD4T cells, resting NK cells, monocytes, M2 macrophages, activated dendritic cells, and resting mast cells (*P* < 0.05) (Figure 3I). The GSVA results demonstrated that the C1 cluster was mainly involved in immune diseases and metabolism-related pathways, such as asthma, drug metabolism other enzymes, glutathione metabolism, porphyrin and chlorophyll metabolism, while the C2 clusters were primarily enriched in cellular conduction-related pathways, such as neurotrophic signaling, the insulin signaling pathway, the GNRH signaling pathway, and ECM receptor interaction (Figure 3J). These findings suggest that, based on the expression of CASCRGs, CAS samples can be divided into two subgroups with significantly different biological functions, especially immune responses.

### 3.3 Identification of common module hub genes

**Figure 4:**
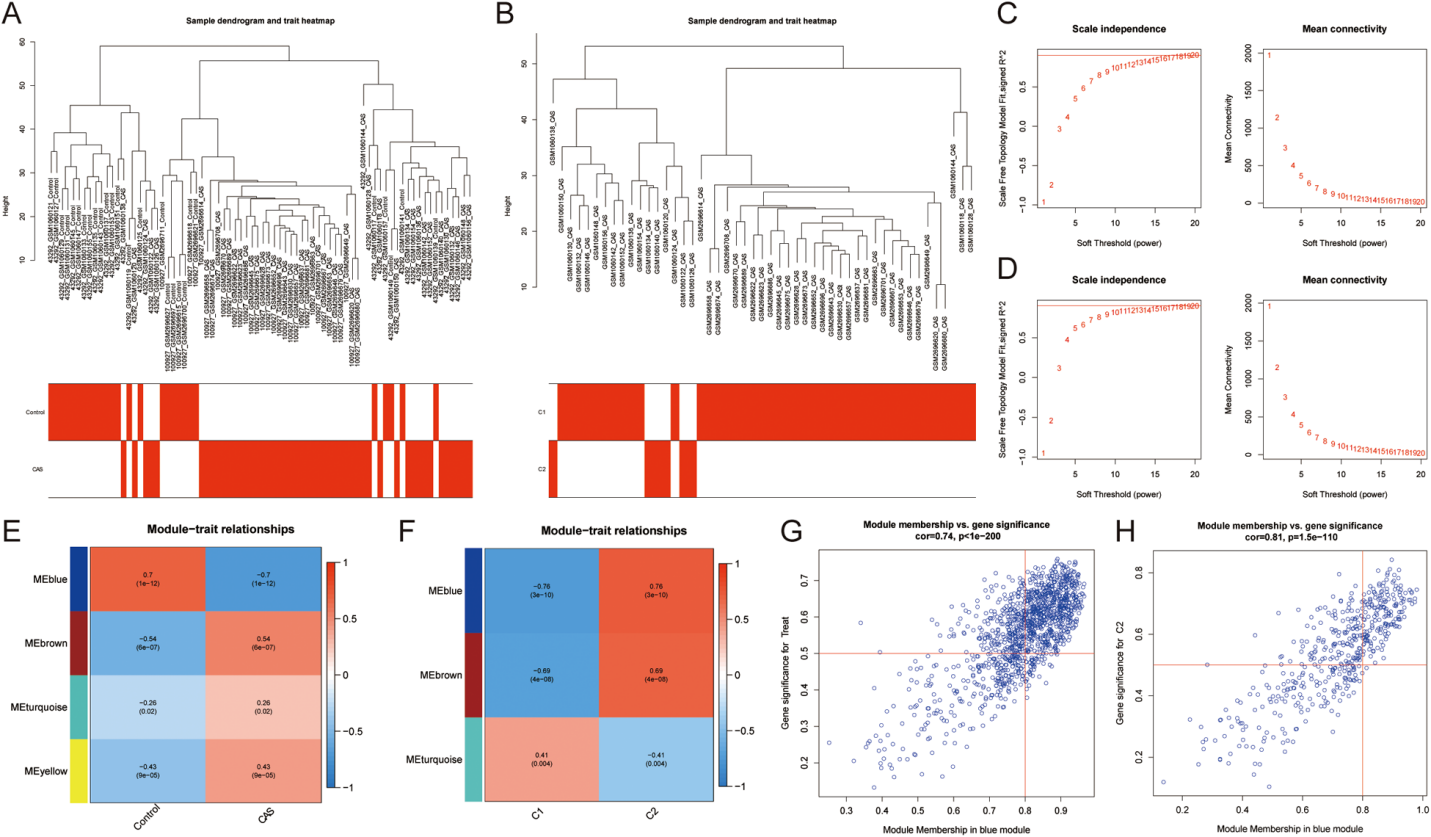
Co-expression network of CAS and cuproptosis-related molecular clusters. **(A,B)** The sample clustering plot of **(A)** all samples and **(B)** molecular clusters after removing outlier samples; **(C,D)** The selection of soft threshold power corresponding to R^2^ = 0.8 of **(C)** all samples (soft threshold = 16) and **(D)** molecular clusters (soft threshold = 15); **(E,F)** Correlation analysis between module eigengenes and clinical status of **(E)** CAS and **(F)** molecular clusters. The blue module was most correlated with CAS traits (correlation coefficient = 0.70, *P* = 1e-12); the blue module was most correlated with the C2 clusters (correlation coefficient = 0.76, *P* = 3e-10). **(G,H)** Scatter plot between module membership in the blue module and the gene significance for **(G)** CAS and **(H)** molecular clusters.

**Figure 5:**
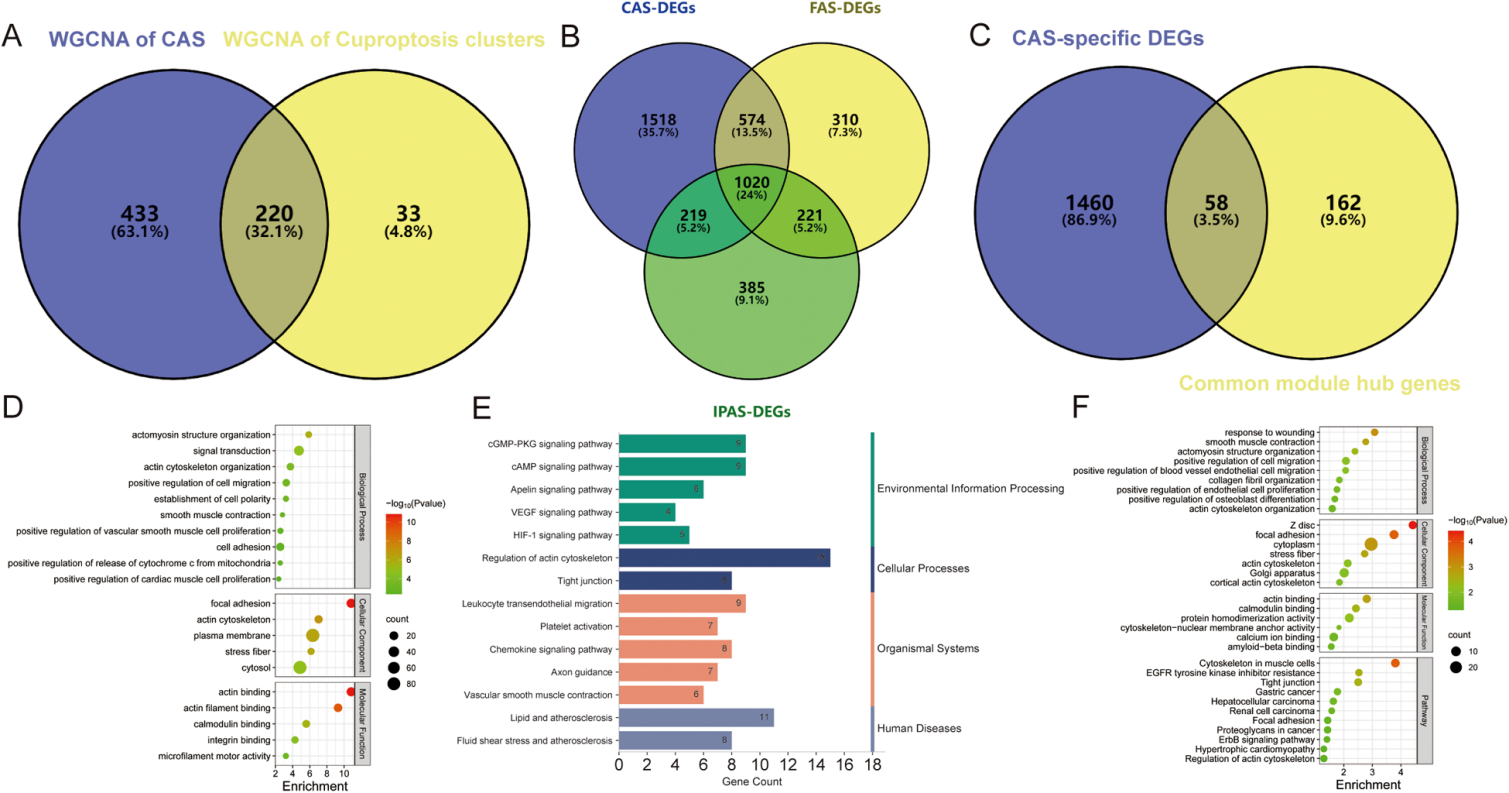
Enrichment analysis of common module hub genes and candidate hub genes. **(A–C)** Venn diagram showing the **(A)** 220 common module hub genes, **(B)** 1,518 CAS-specific DEGs, and **(C)** 58 candidate hub genes; **(D–F)** The biological process, cellular component, molecular function, and pathway analysis of **(D,E)** common module hub genes and **(F)** candidate hub genes.

Co-expression networks and modules were constructed for control and CAS samples using WGCNA (Figure 4A). Setting the soft threshold to 16 resulted in four different colored modules (Figure 4C), with the “blue” module showing the highest correlation with CAS (Figure 4E). This module contained 653 hub genes and was positively correlated with other module genes (Figure 4G). Additionally, WGCNA identified key gene modules associated with CAS and cuproptosis (Figure 4B). Setting the soft threshold to 15 resulted in three different colored modules (Figure 4D). The “blue” modules showed strong correlation with the cuproptosis cluster and were positively correlated with other module genes (Figure 4F). This module contained 253 hub genes and was positively correlated with other module genes (Figure 4H). The intersection of module hub genes from the two “blue” modules resulted in 220 common module hub genes (Figure 5A). Functional enrichment analyses indicate that cuproptosis is involved in the pathogenesis of CAS associated with actin cytoskeleton organzition, cell migration, leukocyte migration across the endothelium, fluid shear stress and atherosclerosis, and platelet activation (Figures 5D,E).

To identify CAS-specific expressed genes, differential expression analysis was performed of carotid, femoral, and popliteal arteries stratified by GSE100927 and obtained 3,331, 2,125, and 1,845 DEGs, respectively, of which 1,518 DEGs were specifically expressed in CAS (Figure 5B), and 58 candidate hub genes were obtained by taking the intersections with 220 common module hub genes, which were CAS-specific expressed differential genes (Figure 5C). Further functional enrichment analysis emphasized the close correlation with biological processes such as vascular smooth muscle contraction, actomyosin structure organization, and cell migration, and are closely related to various cancers, regulation of the actin cytoskeleton, tight junctions, and the HIF-1 signaling pathway (Figure 5F).

### 3.4 Construction and assessment of machine learning models

**Figure 6:**
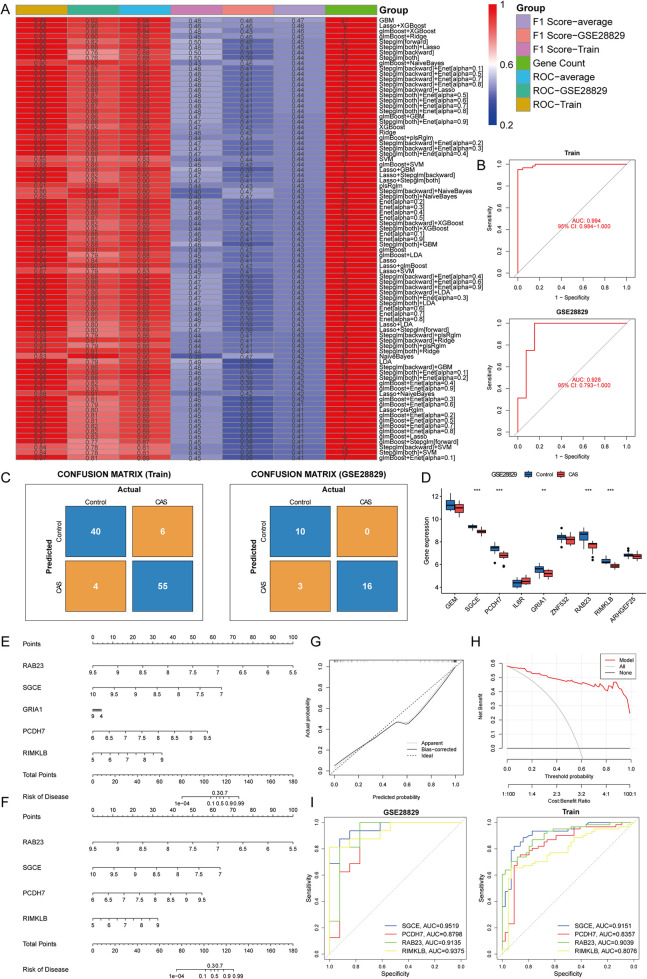
Construction and assessment of machine learning models. **(A)** Construction of 83 multiple machine learning models to screen for hub genes. The best models were screened based on AUC values, F1 scores and gene counts. **(B)** ROC curves for training and validation sets; **(C)** Confusion matrix for training and validation sets; **(D)** A single gene differential analysis was performed in the validation set to screen for hub genes; **(E,F)** Construction of a nomogram for predicting the risk of CAS clusters based on the gene-based Lasso + XGBoost model; **(G,H)** Construction of a **(G)** calibration curve and **(H)** decision curve analysis for assessing the predictive efficiency of the nomogram model; **(I)** ROC curves validate the diagnostic efficacy of the hub gene.

To further screen for cuproptosis-related CAS-specific expressed hub genes, 83 machine learning models were developed for 58 candidate hub genes. Based on the mean AUC value and mean F1 score considered GBM as the best model. However, the GBM model contains 57 genes and too many genes risk overfitting, so the LASSO regression + XGBoost model was chosen (Train: accuracy = 0.905, precision = 0.902, recall = 0.932, F1 score = 0.458; GSE28829: accuracy = 0.897, precision = 1, recall = 0.842, F1 score = 0.457). The analysis revealed that the combination of LASSO regression and XGBoost yielded satisfactory diagnostic efficacy, with an area under curve (AUC) value exceeding 0.9 for both the training and validation datasets, and F1 scores of greater than 0.45 for both the training and validation sets, which contained nine genes, namely, GEM (logFC = −0.90), SGCE (logFC = −0.96), PCDH7 (logFC = −0.91), IL6R (logFC = 0.86), GRIA1 (logFC = −1.02), ZNF532 (logFC = −0.52), RAB23 (logFC = −1.07), RIMKLB (logFC = −0.80), and ARHGEF25 (logFC = −0.71) (Figures 6A,B). The confusion matrices further corroborated the high precision and minimal error rates achieved by these models (Figure 6C). Subsequent single-gene differential expression analysis underscored the differential expression of SGCE, PCDH7, GRIA1, RAB23, and RIMKLB in the validation set and also reduced the risk of overfitting. These genes are down-regulated in CAS and are considered as hub genes (Figure 6D).

To enhance the generalizability of the diagnostic efficacy of hub genes and to delve into the potential gender-specific variations in their expression profiles, a differential expression analysis was conducted on carotid artery samples from GSE100927, stratified by gender. The findings indicated that SGCE, PCDH7, GRIA1, RAB23, and RIMKLB exhibited significant differences in expression levels between male and female samples (*P* < 0.05 & |logFC|>0.5), while no discernible expression disparity was observed between genders within the CAS samples (*P* > 0.05). Consequently, a predictive nomogram was constructed based on hub genes to forecast the prevalence of CAS. However, GRIA1 did not perform well in the nomogram, leading to its exclusion. Subsequently, a revised nomogram was created, incorporating the genes SGCE, PCDH7, RAB23, and RIMKLB for enhanced predictive capabilities (Figures 6E,F). The calibration curves, decision curve analysis, and ROC curves demonstrated that the predictive model of hub genes exhibited excellent diagnostic performance, effectively distinguishing between normal and CAS samples (Figures 6G–I).

The previous study identified four hub genes with generalizability by gender stratification, which can effectively distinguish CAS from normal samples. However, it is also particularly important to distinguish the severity of CAS in the clinic, and the GSE163154 and GSE41571 datasets were used to characterize the expression of hub genes in stable and unstable plaques. The results showed that, compared to stable plaques, the expression of SGCE and PCDH7 was down-regulated in unstable plaque patients (*P* < 0.05 & |logFC|>0.5), whereas there was no difference in the expression of RAB23 and RIMKLB (*P* > 0.05), which suggests that SGCE and PCDH7 may further serve as biomarkers for distinguishing stable and unstable plaque patients.

## 4 Discussion

There is growing evidence that dysregulated copper metabolism and copper excess or deficiency are associated with AS, and extensive research has revealed a correlation between elevated copper levels and cardiovascular disease (36). One study found that copper bioavailability was negatively correlated with carotid intima-media thickness and that CAS was a reliable predictor of early AS in obese patients (37). In addition, Nadina found increased copper levels in CAS plaques, and Nebojša demonstrated that serum copper concentrations also varied in patients with different types of CAS plaques, especially in patients with hemorrhagic plaques of CAS, which were significantly higher than in patients with calcified plaques (38). These findings suggest that elevated copper levels may be involved in the pathogenesis of CAS. Cuproptosis is an emerging mode of cell death triggered by the accumulation of copper ions. Recent research has revealed an association between cuproptosis and the development of various cardiovascular diseases. To investigate the potential mechanism connecting CAS and cuproptosis, four CASCRGs (NLRP3, SLC31A2, CDKN2A, and GLS) were first identified as highly expressed in CAS using differential expression analysis.

There are also some bioinformatics analyses to study the relationship between cuproptosis and CAS; however, because of the differences in the datasets, the conclusions obtained are not entirely consistent. Cui found that FDX1 and SLC3A1 were up-regulated in AS plaques, while GLS was down-regulated, and found that GLS was expressed in vascular smooth muscle cells and SLC3A1 was expressed in macrophages (39). Chen found that SLC31A1 and SLC31A2 were up-regulated in AS plaques, while SOD1 was down-regulated (40). Wang found that ATP7B, MTF1, NLRP3, AOC3, and MT1M were up-regulated in the peripheral blood of AS patients (41). These findings suggest that cuproptosis-related genes may affect AS by regulating copper ion metabolism, oxidative stress, and inflammation (13). However, these studies are specific to systemic AS, and this study was more refined to analyze CAS and showed that cuproptosis-related genes affect CAS by regulating the immune microenvironment, actin cytoskeleton, and cell migration.

Studies have indicated that excess copper ions promote the formation of the NLRP3 inflammasome by inducing ROS production and endoplasmic reticulum stress. The overactivation of the NLRP3 inflammasome is implicated in the onset and progression of AS by mediating inflammatory responses and pyroptosis (25, 42). SLC31A2 is localized in late endosomes and lysosomes, facilitating cellular copper uptake (43). Its high expression in AS is consistent with findings from other studies (40). However, the specific mechanism underlying its association with AS remains poorly studied, warranting further experimental verification. CDKN2A’s association with AS has been confirmed in numerous studies, with high expression observed in AS patients and diseased tissues, significantly correlating with disease severity (44–48). As a recognized modulator locus of AS, CDKN2A may influence the onset and progression of AS by regulating platelet production and reactivity, monocyte and macrophage cell proliferation, and apoptosis (49–51). GLS is an enzyme essential for glutamine catabolism, which protects cells from cuproptosis by promoting glutathione synthesis and reducing ROS damage (52). Our study found that GLS was specifically downregulated in CASCRGs, which may exacerbate AS by affecting macrophage clearance of apoptotic cells, and glutamine catabolism in impaired macrophages. Its expression was significantly down-regulated in patients with CAS plaques, which was also correlated with the severity of clinical adverse events (53, 54).

Previous research has established that cuproptosis promotes the development of AS and contributes to exacerbated oxidative stress, inflammation, endothelial dysfunction, and dyslipidemia (13, 36, 55). In this study, a functional enrichment analysis of the module hub genes revealed that cuproptosis-associated CAS pathogenesis is implicated in biological processes such as vascular smooth muscle contraction, actin cytoskeleton organization, and cell migration. It has been found that a substantial migration of vascular smooth muscle cells into the intima in AS lesions is known to have substantial pro-atherosclerotic effects (56–58). The cytoskeleton, a fundamental structural framework for cell migration, is primarily composed of the actin fiber system (with actin as its subunit) and the microtubule system (with tubulin as its subunit). Research has demonstrated that alterations in the actin cytoskeleton in mouse cells can mitigate AS development by inhibiting the migration of smooth muscle cells (59). In addition, actin remodeling of endothelial cells during leukocyte transepithelial migration is also strongly associated with AS (60). Historically, it was discovered in 1996 that exposure to copper ions can lead to profound disruptions in cellular actin and fibronectin organization. This results in the dissolution of filamentous actin, the disintegration of the actin cortical meshwork, and cytoskeletal morphology alterations (61). Collectively, these findings suggest that cuproptosis might impact the cytoskeleton, thereby disrupting the migration of vascular smooth muscle cells. This disruption could be mediated through the regulation of actin structure, ultimately influencing the progression of AS.

The subsequent results from immune infiltration and correlation analyses indicated that CASCRGs have regulatory effects on multiple immune cells, suggesting that cuproptosis alters the immune infiltration microenvironment in CAS. Cuproptosis, a copper-dependent form of immunogenic cell death, has an intricate relationship with immune responses, which is not yet fully comprehended. Research indicates that cuproptosis might contribute to immune responses through the emission of various damage-associated molecular patterns and tumor-associated antigens (62, 63). Furthermore, cuproptosis can cause cell membrane damage, leading to the release of a significant amount of damage-associated molecular patterns, which effectively stimulate an immune reaction. This reaction not only promotes substantial lymphocyte infiltration but also triggers the secretion of inflammatory cytokines, thereby potentially modifying the tumor microenvironment (64). However, the specific mechanisms by which cuproptosis influences cardiovascular diseases or CAS through immune response modulation are still unclear. It has been demonstrated that a Western diet can initiate NLRP3-dependent inflammatory responses, induce the proliferation and reprogramming of myeloid progenitor cells, and affect innate immune reprogramming (65). Moreover, inhibiting NLRP3 activity has been shown to reduce the M1/M2 macrophage ratio, prevent macrophages from transitioning to the pro-inflammatory M1 phenotype, and decrease the levels of pro-inflammatory cytokines such as IL-6, IL-1β, and TNF-α (66, 67). Additionally, variations in CDKN2A expression have been linked to the regulation of T cell phenotypes in AS and type 2 diabetes. Lower CDKN2A expression levels have been associated with higher levels of CDK4, and the use of a CDK4 inhibitor has been shown to increase the levels of Treg cells and the activation of the transcription factor phospho-STAT5 (reference 30176239). Decreased expression of CDKN2A/2B/2BAS in leukocytes has also been correlated with an increase in proatherogenic CD14++CD16 + monocytes (68). Lastly, glutaminase-1-mediated glutaminolysis plays a crucial role in facilitating macrophage clearance of apoptotic cells during homeostasis in mice. Impaired macrophage glutaminolysis can exacerbate atherosclerosis, and glutaminase-1 expression has been strongly linked to atherosclerotic plaque necrosis in patients with cardiovascular disease (53). The above studies suggest that the cuproptosis-related genes NLRP3, CDKN2A, and GLS regulate the transformation of T cell, macrophage, and monocyte phenotypes and alter the immune microenvironment to some extent.

In addition, the immune infiltration analysis revealed that CAS had a lower abundance of monocytes, which is consistent with other bioinformatics analysis studies on AS (69–71). AS is considered a chronic inflammatory disease and monocytes are considered pro-inflammatory cells, and studies have shown that monocytes, once they enter the diseased blood vessel, differentiate to become macrophages and release more inflammatory factors through the uptake of modifying lipoproteins such as ox-LDL, forming foam cells, which may account for the lower abundance of monocytes in CAS tissue (72).

Subsequently, the application of 83 machine learning algorithms was employed to identify hub genes for cuproptosis-related CAS. The Lasso regression combined with the XGBoost model emerged as the most effective, demonstrating the highest AUC values. Further external independent validation through single-gene differential analysis revealed SGCE, PCDH7, RAB23, and RIMKLB as hub genes. These genes modulate alternative splicing by influencing cytoskeleton composition and cell adhesion and are found to be downregulated in CAS. Further validation of the generalizability of the hub genes was conducted by stratifying the GSE100927 dataset across both genders. To investigate whether these genes could differentiate the severity of CAS, validation was extended to two additional datasets, which demonstrated that the SGCE and PCDH7 genes were down-regulated in unstable CAS plaque patients compared with stable CAS plaque patients, suggesting their potential as biomarkers for distinguishing stable from unstable plaques in CAS.

SGCE is a component of the sarcoglycan complex that forms a link between the F-actin cytoskeleton and the extracellular matrix, and defects in its components have been shown to be associated with myoclonus-dystonia syndrome and cardiomyopathy (73, 74). It was found that patients with sarcoglycan deficiency may have a molecular basis for differential smooth muscle dysfunction that induces coronary vasospasm, affecting AS by influencing endothelial dysfunction and arterial remodeling (75–77). In addition, SGCE affects carotid biomechanical characteristics; SGCE-deficient mouse carotid arteries had decreased distensibilities in pressure-diameter tests and generated elevated axial loads and stresses in axial force-length tests (78).

PCDH7 is a crucial integral membrane component of the calreticulin superfamily, which is instrumental in modulating the dynamics of intercellular adhesion and the structural integrity of the contractile actin cytoskeleton (79). Recent investigations have revealed that PCDH7 expression is significantly diminished in atherosclerotic lesions of the monkey iliac artery, as well as in atherosclerotic plaques (80). Intriguingly, it has been established that the preservation of intercellular adhesion serves to curb the proliferation of vascular smooth muscle cells, a pivotal process in the progression of AS (81).

RAB23 encodes a small GTPase, which functions as a negative regulator of the Sonic hedgehog signaling pathway. It plays a crucial role in transporting transmembrane receptors related to Sonic hedgehog signaling to the cilia (82, 83). The vesicular trafficking of RAB23 is vital for the formation and composition of cilia, which are sensitive to shear forces. Primary cilia have been shown to inhibit the progression of AS by triggering calcium influx, activating endothelial nitric oxide synthase, and promoting nitric oxide production, thereby reducing vascular calcification and protecting endothelial function from blood flow disturbances (84). In addition, statins are commonly used as drugs for the treatment of CAS, and pravastatin was found to significantly down-regulate the level of RAB23 in patients, which may improve lipid metabolism and inhibit cholesterol biosynthesis by regulating the Sonic hedgehog pathway, thus treating AS (85–87).

RIMKLB facilitates the production of β-citrullinyl-L-glutamate and N-acetyl-L-aspartyl-L-glutamate, both of which play pivotal roles in amino acid metabolism. Recent studies have identified the role of RIMKLB in maintaining the balance of zinc and copper ions within the epicardial adipose tissue during heart failure (88). However, the connection between RIMKLB and the development of AS remains to be fully elucidated.

In this study, a comprehensive bioinformatics analysis was conducted to explore the relationship between CAS and cuproptosis. Four CASCRGs (NLRP3, SLC31A2, CDKN2A, and GLS) were identified, revealing that cuproptosis is intimately linked to immune responses and can significantly alter the immune infiltration microenvironment within CAS. Two distinct clusters of cuproptosis-related molecules were discerned within CAS samples, exhibiting notable variances in immune responses. Multiple models were developed based on machine learning techniques, with the Lasso regression combined with the XGBoost model showing satisfactory diagnostic efficacy. Furthermore, four hub genes (SGCE, PCDH7, RAB23, and RIMKLB) were identified and used to construct a predictive nomogram for the incidence of CAS. Additionally, the genes SGCE and PCDH7 were found to be effective in assessing the stability of CAS plaques.

However, this study has some limitations. Firstly, the datasets used in this study were sourced from different countries, such as France and New Zealand, which may introduce bias and make the findings of this study more applicable to Western countries. Additionally, the datasets from various platforms may have omitted some potential DEGs after merging. Secondly, due to database limitations, it was not possible to obtain more CAS samples for the study. The relatively small sample size hindered the effectiveness of unsupervised cluster analysis in subtyping group CAS, potentially leading to unstable groupings or the omission of certain subtypes. Finally, this study did not perform experimental validation of CASCRG in CAS models, neither *in vivo* nor *in vitro*, and the mechanism by which cuproptosis influences CAS through immune response modulation remains unclear.

## Conclusions

Cuproptosis alters the CAS immune infiltration microenvironment and may regulate actin cytoskeleton formation.

## Data Availability

The original contributions presented in the study are included in the article/Supplementary Material, further inquiries can be directed to the corresponding authors.
